# Controlled ovarian hyperstimulation and intrauterine insemination cycles in patients with unilateral tubal blockage diagnosed by hysterosalpingography 

**Published:** 2011

**Authors:** Mahbod Ebrahimi, Firoozeh Akbari Asbagh, Azizeh Ghaseminejad

**Affiliations:** Department of Obstetrics and Gynecology, Mirza Koochak Khan Hospital, Faculty of Medicine, Tehran University of Medical Sciences, Tehran , Iran.

**Keywords:** *Hysterosalpingography*, *Intrauterine insemination*, *Ovarian hyperstimulation*

## Abstract

**Background:** Controlled ovarian hyperstimulation and intrauterine insemination (IUI) cycle is an ideal protocol for some subfertile patients. So, we decided to try this therapeutic protocol for the patients with unilateral tubal blockage diagnosed by hysterosalpingography (HSG).

**Objective: **To evaluate the effect of unilateral tubal blockage diagnosed by HSG on cumulative pregnancy rate (CPR) of the stimulated IUI cycles.

**Materials and Methods: **A cross-sectional analysis was performed between October 2006 and October 2009 in an academic reproductive endocrinology and infertility center. Two groups of patients undergoing stimulated IUI cycles were compared. Sixty-four infertile couples with unilateral tubal blockage diagnosed by HSG as the sole cause of infertility in the group (І), and two hundred couples with unexplained infertility in the group (II). The patients underwent 3 consecutive ovarian hyperstimulation (Clomiphen citrate and human menopausal gonadotropin) and IUI cycles. The main outcome measurements were the CPRs per patients for 3 consecutive stimulated IUI cycles. **Results:** Cycle characteristics were found to be homogenous between the both groups. CPRs were similar in group І (26.6%) and group II (28%) (p=0.87; OR=1.075; 95% CI: 0.57 -2.28).

**Conclusion:** Unilateral tubal blockage (diagnosed on HSG) has no effect on success rate of stimulated IUI cycles, so COH and IUI could be recommended as the initial therapeutic protocol in these patients.

## Introduction

Tubal pathology ranks among the most frequent causes of subfertility, next to ovulatory disorders and sperm defects (1). Therefore, assessment of tubal patency is a fundamental part of infertility workup (2). Investigation for tubal disease can be done by radiological tests [hysterosalpingography (HSG), selective salpingography and hystero-contrast-sonography (HyCoSy)], microbiological tests (Chlamydia testing of the serum or urine) and surgical tests (laparoscopy with chromo-pertubation, falloscopy and fertiloscopy) (3). Diagnostic laparoscopy is generally accepted as the most accurate procedure to detect tubal pathology and periadnexal adhesions (3), owing to the noninvasive nature and low cost. HSG is widely used as a first-line approach to assess uterine anatomy and tubal patency in routine infertility workup (4). HSG has 65% sensitivity and 83% specificity for tubal obstruction (5). In addition good correlation between HSG and laparoscopy regarding tubal patency has been demonstrated (6-8).

There is a growing tendency to bypass diagnostic laparoscopy in couples with a normal HSG (patent tubes) who will undergo intrauterine insemination (IUI) treatment for unexplained infertility (UEI), mild male subfertility and cervical hostility (9-11). Management of the patients with unilateral tubal blockage diagnosed by HSG is a controversial subject. Although, a number of reports have recommended laparoscopy to confirm followed by the diagnosis (3, 12, 13), reconstructive tubal surgery by laparoscopy, selective salpingography and tubal catheterization (SS/TC) or hysteroscopic transcervical tubal cannulation (3, 13-15). The other practitioners suggested that one-sided tubal pathology does not influence the possibility of treatment independent pregnancy (16). They recommended that laparoscopy may be omitted in women with normal HSG or suspected unilateral tubal pathology on HSG, since it did not change the original treatment plan in 95% of the patients (10). In addition, they showed that bilateral tubal pathology diagnosed at HSG or laparoscopy affect fertility prospects strongly, whereas unilateral pathology affect future fertility less severely (17, 18). Therefore controlled ovarian hyperstimulation (COH) and IUI is recommended as the initial treatment of choice in patients with unilateral tubal occlusion diagnosed by HSG (19). So far, only one retrospective study has tried to evaluate pregnancy rates after COH and IUI in women with HSG findings suspicious to unilateral tubal occlusion (19). Thus we decided to carry out a prospective study to assess the effect of unilateral tubal blockage on success rate of COH and IUI in these patients. 

## Materials and methods

**Patients**

Subjects were sub-fertile couples who were referred to reproductive endocrinology and infertility center (Mirza Koochak Khan Hospital, Tehran, Iran), between October 2006 and October 2009. Approval from the local institutional Ethics Committee (Tehran University) was given before starting the study. The Patients were informed about the purpose and hazards of this study. 

The present analysis was limited to couples with following criteria: female partner age ≤35 years, regular menstrual cycles with mid luteal progesterone >10 ng /m L, basal FSH and LH<10 IU/ L, normal early follicular phase ultrasound, no endocrine abnormalities, and normal semen analysis according to World Health Organization (20). HSG was performed in these patients, as part of routine infertility work-up, shortly after the menstrual period. Water –soluble contrast medium (Visipaque™) was used. 

According to HSG findings, the patients were divided into two groups. The group (І) included patients with normal uterine cavity, normal transfer and spill of contrast medium from one fallopian tube, and the group (II) included patients with normal uterine cavity and bilateral patent tubes. The patients with uterine cavity abnormalities or/and bilateral blockage were excluded from the study. In the next menstrual cycle after HSG taking, treatment protocol was started. The patients in both groups underwent 3 consecutive cycles of ovarian hyperstimulation and IUI. All cycles were gently stimulated with 50 mg oral tablets of clomiphen citrate (clomiphen citrate, Iran hormone, Tehran, Iran) twice daily for 5 days starting on day 3 of the menstrual cycles and starting dose of 75 IU human Menopausal Gonadotropin (Menogan, Ferring, Germany) on 7-9 days of the cycles. The dosage of human Menopausal Gonadotropin (hMG) was adjusted according to the ovarian response. Stimulation continued until one to three follicles reached mean diameter of 18 mm, then 5000 IU hCG (Profasi, Serono, Geneva, Switzerland) was given, and the single IUI was performed 36 hours later. Monitoring of cycles by E _2 _was done in selected patients with hyper-response. Cycles with more than three dominant follicles and/or serum E_2 _level >1500 pg/m L were canceled to avoid ovarian hyperstimulation syndrome and high-order multiple pregnancy. Serum β-hCG was requested 2 weeks after hCG administration, and intrauterine pregnancy was confirmed by detection of a gestational sac using transvaginal ultrasound 4 weeks after insemination. If pregnancy did not happen, this protocol was repeated two times consecutively. The main end-point of this study was pregnancy rate in these three consecutive cycles.

**Statistical analysis**

The statistical program for Social Sciences (SPSS, version 16; SPSS, Chicago, IL) was used for statistical analysis. Demographic data and cycle characteristics of both groups were expressed as mean SD for continues data and proportional rate for categorical data. Comparison of continues data was performed by Student’s t-test, categorical data by univariable logistic regression analysis and chi-squared test, and data with percentage value by Mann-Withney U test. Comparison of cumulative pregnancy rates between the two groups were performed by Chi-squared test. Odd ratio (OR), 95% Confidence interval (95 %CI) and corresponding p–value for these data were estimated by univariable logistic regression analysis. P-value<0.05 was considered to be statistically significant. 

## Results

During the study period, data on 289 couples were collected. Twenty-five patients (8.65%) dropped out for various reasons. Of these 25 patients, 7 (2.42%) were in group (І) and 18 (6.23%) were in group (II). We excluded these patients from the study. Finally, we compared data on 264 patients. Sixty-four couples were in group (І), and 200 couples were in group (II). In group (І), 39 women had proximal part tubal blockage, and 25 women had mid/distal blockage. Seven cycles were canceled for ovarian hyperresponse, and these patients were treated in their further cycles with lower dosage of gonadotropins. Demographic characteristics of the two groups are shown in (Table I). There were no differences between the two groups in age, parity, duration of infertility, basal FSH, LH, E_2 _level and sperm parameters. There were statistically significant higher rate of previously diagnosed PID and extrauterine pregnancies in group (I) (Table II). Cycle characteristics of two groups are shown in (Table II). There were no differences between two groups regarding cycle characteristics. Of 711 cycles, a total of 73 pregnancies occurred; 17(26.6%) pregnancies were in group (І), and 56(28%) pregnancies in group (II). In each group one pregnancy was tubal type of extra uterine pregnancy. None of the patients developed ovarian hyperstimulation syndrome or pelvic inflammatory disease. The cumulative pregnancy rates per patient after 3 cycles of COH and IUI were (17/46) 26.6% in group (І) and (56/200) 28% in group (II) (p=0.87; OR =1.075; 95% CI: 0.57-2.28).

**Table I:** Demographic data of the patients in group І (unilateral tubal blockage) and group II (UEI).

	**Group І**	**Group II**	**p-value** **OR (95% CI)**
No. of couples	64	200	
No. of cycles	170	541	
Female age	27.143.34	28.133.76	0.06
Male age (years)	33.34.47	33.44.97	0.88
Parity	0.40.23	0.30.15	0.32
Duration of infertility (years)	4.332.66	4.842.65	0.183
Basal FSH (IU/ L)	5.842.2	6.112.O3	0.35
Basal LH (IU/ L)	4.932.54	4.842.18	0.79
Basal E_2 _(pg/m L)	31.6117.42	35.4514.06	0.075
Basal sperm count ( 10^6 ^/ m L)	64.2231.82	61.0823.7	0.74
Basal total Sperm motility (%)	60.8811.07	63.6711.56	0.091
Basal total normal sperm morphology (%) (WHO criteria)*	47.1913.46	45.7615.65	0.48
Previous EUP* *	5 (7.8%)	3 (1.5%)	0.021****
Previous diagnosed PID***	8 (12.5%)	10 (5%)	0.045****

Independent student’t test (continues data).

Mann Withney U test (percentage data), Univariable regression analysis, Chi-square test (categorical data).

* =World Health Organization,

** =Extrauterine pregnancy,

*** = Pelvic inflammatory disease.

**** =Statistically significant difference: < 0.05.

**Table II:** Cycle characteristics of the patients in group І(unilateral tubal blockage), and group II (UEI).

	**Group І**	**Group II**
No. of dominant follicle > 16 mm	2.090.74	1.91079
Endometrial thickness on the day of HCG	9.30.4	9.20.6
Total progressive motile sperm number after sperm preparation (10^6 ^/m L)	39.123.4	43.831.1
Total amount of gonadotropins ( IU)	330.86167.96	290.63100.01

Independent student’s t-test.

* p- values of data are non-significant.

## Discussion

Here, we report result from a cross-sectional study assessing the effect of unilateral tubal blockage (diagnosed by HSG) on CPR of COH and IUI. There was no statistically difference in CPRs between group (І) with unilateral tubal blockage on HSG as the sole abnormal parameter in their infertility investigation and group (II) with UEI and normal HSG findings (26.6% and 28%, respectively) (p=0.87; OR =1.075; 95% CI: 0.57-2.28).

Traditionally, when possibility of tubal occlusion has been shown by HSG, laparoscopy is suggested as a mandatory step to confirm or preclude the existence of this pelvic pathology as the cause of infertility (21, 22). After confirming tubal occlusion, reconstructive tubal surgery or trans cervical catheterization is the next step (3, 13-15). Nevertheless laparoscopy values for its accurate and extensive diagnostic and therapeutic capabilities, it should be recognized that laparoscopy is an invasive procedure, involving risks of general anesthesia, vascular and gasterointestinal accident, pain and discomfort (23). 

It is an expensive tool, especially when the economical resources do not allow the acquisition of sophisticated medical equipment. The additional information provided by diagnostic laparoscopy is useful only to the extent that laparoscopic findings would change the management of an infertile couple. Lavy *et al* reported, out of the 63 patients with normal HSG or suspected unilateral tubal pathology, only three women (4.7%) had abnormal laparoscopic findings that mandated a change in the original treatment regimen. So they recommended that laparoscopy is not indicated in these patients with unilateral tubal pathology on HSG, and the same treatment protocol intended for patients with normal HSG may be applied to this sub-group of patients, under the definition of unexplained infertility (10). 

We should in mind, that HSG is a low cost procedure with high specificity (5).The additional value of HSG is particularly the assessment of the uterine cavity (24). Uterine cavity malformations with a frequency of 10-15% in infertile women can be visualized by HSG, although the effectiveness of treatment of uterine abnormalities on improving pregnancy rate has not been established (2). Although, several reports have documented the shortcomings of HSG in establishing the diagnosis of peritubal adhesions, and minimal and mild endometriosis (25), it is still a matter of debate whether such lesions affect fertility and whether treatment of these lesions results in higher pregnancy rates (26, 27).

Chlamydia antibody test (CAT) is a simple and inexpensive test method, and good meta-analysis has suggested that the discriminative capacities of Chlamydia antibody titer and HSG in the diagnosis of any tubal pathology are comparable (28). Unfortunately, Chlamydia antibody test (CAT) fails to provide information about the severity of tubal pathology, which is important for fertility prognosis and treatment .Furthermore; it cannot detect tubal pathology due to other causes. 

Falloscopy and fertiloscopy are highly specialized techniques for the assessment of status of the fallopian tubes. “These procedures are not widely practiced and further research should ascertain their values as part of the routine investigation of tubal diseases” (3). 

We demonstrated that obstruction of one fallopian tube (diagnosed by HSG) did not significantly affect the incidence of pregnancy in stimulated IUI cycles, compared with patients with UEI and patent tubes (p=0.87; OR=1.075; 95% CI: 0.57-2.28). Our findings could be explained in 3 ways: 

1/ it is possible that obstruction of one tube plays a minor or no role in sub-fertility (29). 

2/ proximal tubal obstruction was the major cause of tubal blockage in our study (proximal vs. mid/distal obstruction, 60.94% and 39.06%, respectively). Non filling of proximal part at the time of HSG can sometimes be due to reversible causes such as: the presence of mucus, polyps, intra mural debris or amorphous material and tubal spasm (3, 15). Dessole *et al* found in patients with tubal obstruction diagnosed by HSG, that second conventional HSG after one month achieved bilateral tubal patency in 60% of patients. So they suggested that modification of both, the tubal peristaltic activity and the intraluminal biochemical environment, related to the cyclic hormonal changes, can spontaneously remove the mucus and amorphous material (30).Therefore the tubal blockages shown by HSG could be a temporary events. 

3/ HSG is a useful tool for evaluating tubal patency, that does not necessary equate satisfactory tubal function. It is impossible to determine the intricate physiological function of the fallopian tube just by a simple test such as HSG (3). So in many cases with bilateral patent tubes on HSG, tubal function might be unsatisfactory.

 Mol *et al* suggested that “ a completely normal HSG or a HSG with one-sided abnormality affects fertility prospects slightly, so they recommended that laparoscopy can be postponed until at least 10 months after a normal or one-sided abnormal HSG, whereas laparoscopy provides useful information, immediately after a two-sided abnormal HSG” (29). On the other hand, delaying laparoscopy after a certain number of unsuccessful IUI cycles could lead to a decrease of the total number of laparoscopies performed, and the probability of finding clinically relevant abnormalities by laparoscopy could be higher, because patients without intra abdominal pathology would already become pregnant before laparoscopy.

Farhi *et al* found similar CPRs in 62 women with unilateral tubal occlusion (diagnosed by HSG) and 115 patients with UEI. In subgroup of these women with mid/distal occlusion of a single tube the success rate was rather than this in women with UEI (19% and 42.6%, respectively) (p=0.044; OR=0.31; 95% CI=0.1-1.0) (19).The flaw of these results is limited number of women , who had mid/distal obstruction in the study . So, the results must be interpreted with caution, and we need better studies with larger sample size for confirmation of these results (19). 

The selection bias hampers the interpretation of findings, when comparing CPRs between the patients with unilateral tubal obstruction and the patients with UEI, Because, UEI is a condition, that might be associated with unknown fertility reducing factors. So, the results must be interpreted with caution. However, these results were comparable to the results that were provided in the other studies (10, 19).

Follow-up of our study ended after 3 cycles of IUI. The subsequent obvious question concerns what the next step should be taken after 3 unsuccessful IUI cycles. Should this be diagnostic laparoscopy and/or reconstructive tubal microsurgery or IVF, or is continued IUI cycles still an option? However, in our ward, next strategy is indeed diagnostic or/and therapeutic laparoscopy and if needed reconstructive tubal microsurgery. But as far as we are aware, there are no published studies on this subject. Prospective studies should be performed to determine the effect of laparoscopic intervention on the pregnancy rate after unsuccessful IUI cycles.

In conclusion: on the basis of these findings, HSG can be suggested as the less invasive procedure to assess tubal patency and recommended that unilateral tubal pathology (diagnosed by HSG) has no significant effect on success rate of IUI cycles, and controlled ovarian hyperstimulation and IUI in 3 consecutive cycles is the successful approach in the patients with suspicious to unilateral tubal pathology..
